# Effect of Different Antioxidants on the Quality of Smoked and Air-Dried Top Mouth Culter

**DOI:** 10.3390/foods15111889

**Published:** 2026-05-27

**Authors:** Yujie Lei, Xiaomei Gao, Wei Yu, Yu Qiao, Sha Cai, Xin Li

**Affiliations:** Hubei Key Laboratory of Characteristic Resources and Utilization, Institute of Agro-Products Processing and Nuclear-Agricultural Technology, Hubei Academy of Agricultural Sciences, Wuhan 430064, China; yujielei2022@sjtu.edu.cn (Y.L.); gaoxiaomeisy@126.com (X.G.); qiaoyu412@sina.com (Y.Q.); 378079021@163.com (S.C.)

**Keywords:** topmouth culter, smoking, air drying, tea polyphenols, tebutylhydroquinone, biogenic amines

## Abstract

To improve the fish quality of air-drying topmouth culter, this study was carried out to add 0.2 wt% of antioxidants (tebutylhydroquinone (TBHQ) and tea polyphenol (TP)) in combination with salt to salinate topmouth culter and to investigate the effect of antioxidants on the quality as well as structural characteristics of topmouth culter during air-drying at 25 °C for 24 h. The water content of the fish ranged from 63.45% to 66.01% when air-dried for 24 h. During air-drying, the water content decreased by 10%, water activity decreased, and the proportion of bound water increased slightly. The loss of water in the fish led to a dense structure and a significant increase in firmness and chewiness. The air-dried fish had reduced brightness and increased redness and yellowness. In addition, the results showed that the addition of 0.2 wt% of TP and/or TBHQ reduced the chemical spoilage of salted air-dried fish, as reflected in total volatile basic nitrogen (TVBN), thiobarbituric acid-reactive substances (TBARS), and total viable count (TVC) and biogenic amine content, thus maintaining the quality of the fish meat. This study can provide a theoretical basis and value for the practical use of antioxidants in salted air-dried topmouth culter.

## 1. Introduction

Topmouth culter (*Culter alburnus*) is a freshwater carnivorous fish of the carp family, which is popular in the Chinese aquatic market because of its good taste and high nutritional value [1]. But because of its high moisture content, rich nutrients and microorganisms carried by it, the fish is highly susceptible to spoilage [2]. Salting, smoking and drying are generally used to extend the shelf life of the fish [3]. Solar drying is a low-cost technique for preserving fish. The traditional air-drying method is mainly sunlight, and this technique has problems such as uncontrollable temperature and the possibility of dust and other foreign objects being brought in [4,5]. In recent years, cold air drying, hot air drying, microwave drying, freeze drying, solar drying and convection air drying have been widely used in fish processing [6,7,8,9]. The drying process involves the transfer of heat to promote dehydration of aquatic products, reduction in moisture content and water activity to slow down the growth of spoilage microorganisms and decrease in chemical and enzymatic reaction rates. The final quality of fish products is influenced by the drying method. Previous studies have shown that cold air drying maximizes the inhibition of fat oxidation and reduces protein denaturation in fish, allowing for maximum retention of fish nutrients [10,11]. However, cold air drying suffers from a long drying cycle, and this drying method cannot completely inhibit the oxidative denaturation of fats and proteins and the reproduction of spoilage microorganisms.

To prolong the shelf life of fish and ensure its freshness, antioxidants are widely used in the storage and preservation of aquatic products, as antioxidants can effectively reduce the oxidation of meat products and delay the generation of off-flavors [12,13]. It should be noted that the correct use of synthetic antioxidants, within the limits of the regulated acceptable daily intake (ADI), does not pose a risk to consumer health. Regulatory agencies such as the FAO/WHO have established safety guidelines for synthetic antioxidants in food preservation. Conversely, natural antioxidants are not inherently free from potential harm; excessive intake of certain natural antioxidants, such as vitamins or carotenoids, may also lead to adverse health effects. Therefore, both synthetic and natural antioxidants should be used judiciously, with attention to their respective safety thresholds. The main mechanism of antioxidant action is that antioxidants provide hydrogen radicals, which react with free radicals to form relatively stable inactive products to achieve the effect of delaying lipid oxidation [14]. Due to the structural differences in antioxidants, their specific working mechanisms and effects are different. Synthetic antioxidants have been widely used in the food industry for many years, but due to their potential toxicity and carcinogenicity, natural antioxidants have become more acceptable to consumers in recent years [15,16]. Feng, Ng, Mikš-Krajnik and Yang explored antimicrobial edible coatings of gelatin extracted from fish skin and fish bones and tea polyphenols to inhibit the deterioration of fish filets during refrigeration [17]. Alexandre et al. compared the effects of sodium tripolyphosphate, sodium ascorbate, sodium isoascorbate and natural antioxidants (green tea extract and propolis extract) on the physicochemical properties, including composition, lipid oxidation, pH, water activity and color of tilapia stored at −18 °C for 6 months [18]. The results showed that sodium tripolyphosphate, sodium isoascorbate and ascorbic acid reduced lipid oxidation during frozen storage of tilapia; however, green tea and propolis extracts were not effective in controlling lipid oxidation.

The aim of this study was to investigate the effect of antioxidants (tert-butylhydroquinone and/or tea polyphenols) on the quality of topmouth culter during air drying at 25 °C. The topmouth culter was treated with 0.2 wt% antioxidant and 2 wt% salt, then air-dried for 24 h. Subsequently, the moisture content, water activity and water distribution of the fish were determined to understand the water status during air drying. The total volatile base nitrogen (TVBN), thiobarbituric acid–reactive substance (TBARS), total viable count (TVC) and biogenic amine content of the fish were used to evaluate the effect of antioxidants. In addition, the changes in color, texture and microstructure of fish meat with air-drying time were monitored. This work can provide a theoretical basis for the application of antioxidants in the factory processing of topmouth culte to further improve the quality of air-dried fish meat.

## 2. Materials and Methods

### 2.1. Materials

Fresh topmouth culter (*Culter alburnus*), weight 750 g ± 50 g, length 40 cm ± 2 cm, were purchased from Wushang supermarket, Wu Han, Hubei, China. Live fish were eviscerated from the back and de-scaled by market vendors. The samples were transported to the laboratory within 30 min.

Plate count agar (PCA) was purchased from Qingdao Hi-Tech Industrial Park Haibo Biotechnology Co., Ltd. (Qingdao, China). Dansulfonyl chloride (purity > 98%, HPLC) was purchased from Shanghai Macklin Biochemistry Co., Ltd. (Shanghai, China). Biogenic amine standard (HPLC) was purchased from Tianjin Alta Technology Co., Ltd. (Tianjin, China). Other reagents with analytical grade were purchased from the Sinopharm Chemical Reagent Co., Ltd. (Shanghai, China). Deionized water was used in this experiment.

### 2.2. Pre-Processing, Salting and Drying of Topmouth Culter

Topmouth culter were rinsed with tap water and placed on a stainless-steel wire mesh to drain for 20 min. Then, the fish were randomly divided into four groups. The first group was dry salted with 2.0 wt% salt without antioxidants as the control group, and the other three groups were salted with 2.0 wt% salt mixed with 0.2 wt% tert-butylhydroquinone (TBHQ), 0.2 wt% tea polyphenol (TP), and 0.2 wt% complex antioxidants (0.1 wt% TBHQ and 0.1 wt% TP), respectively. The fish filets were cold-smoked at 25 °C for 2 h using apple wood chips. All samples were placed flat from the back open in the smoker house (BYXX-50, Jiaxing EXPRO Industrial Co., Ltd., Jiaxing, China) and dried at 25 ± 2 °C using low air speed for 0 h, 6 h, 12 h, 18 h and 24 h, respectively.

### 2.3. Water Content and Water Activity

Referring to the method described by Yu et al., the water content of the samples was determined by the direct drying method [11]. Briefly, 5 g of the fish sample was mixed with 10 g of fine-grained sea sand at 105 °C, and the mass of the dried sample was recorded. The determination was carried out in triplicate.

The water activity of the samples was determined using the LabMaster-aw moisture activity meter (Novasina, Lachen, Switzerland). The churned sample was placed evenly in the moisture activity measuring chamber. The measurement temperature was 25 °C. The average value was taken in five samples.

### 2.4. Low Field Nuclear Magnetic Resonance (LF-NMR)

According to the protocol of Shao et al., the water distribution of fish meat was determined by LF-NMR [19]. Samples with a size of 1.5 cm × 1.5 cm × 1.0 cm were placed in the 30 mm diameter NMR glass tube. The relaxation times (T2) of water molecules were analyzed using the Carr-Purcell-Meiboom-Gill (CPMG) sequence. The transverse relaxation time and area were determined by using the MultiExp Inv Analysis software (Version 2.0, Niumag Co., Ltd., Shanghai, China). The main parameters were set to a waiting time (TW) of 3000 ms, an echo time (TE) of 0.5 ms, a number of echoes (NECH) of 1000, and a number of scans (NS) of 4. Each sample was measured in triplicate.

### 2.5. Color Measurement

Following the procedure described by Ortiz, Lemus-Mondaca & Vega-Gálvez, the color of fish muscle was determined using a spectrophotometer (CS-580A Caipu, Guangzhou, China), including L* (brightness), a* (redness) and b* (yellowness) values [20]. The calibration was performed with the standard blank version, and the average value was taken for each group of 5 samples.

### 2.6. Texture Analysis

As described by Pankyamma et al., the fish was cut into 1.5 cm × 1.5 cm × 1.0 cm pieces and the texture properties were determined using a texture analyzer (Texture Technologies Corp., Scarsdale, NY, USA) [21]. The P36/R model was selected, and the textural properties of the fish were determined in TPA mode at room temperature. The test parameters were: 5 mm/s pre-test and post-test velocity, 1 mm/s test velocity and 5 g trigger force. The tests were performed five times.

### 2.7. Total Volatile Basic Nitrogen

The total volatile base nitrogen (TVBN) content of the samples was determined according to the method described by Bekhit et al. with slight modifications [22]. 10 g of chopped fish was mixed with 100 mL of distilled water, homogenized at 8000× rpm for 2 min with a FSH-2A homogenizer, and then filtered. 10 mL of filtrate and 5 mL of magnesium oxide solution (10 g/L) were added sequentially to the micro Kjeldahl distillation apparatus. The mixture was heated, and 10 mL of boric acid solution (20 g/L) and 5 drops of Tashiro indicator (1 g/L methyl red ethanol solution mixed with 1 g/L hypomethyl blue ethanol solution in a 2:1 volume ratio) were used as the receiving solution to continuously receive the distillate for 5 min, and then the lower end of the condenser tube was lifted off the distillate receiving bottle and distilled for 1 min. The receiving solution was titrated with 0.01 M HCl. The total volatile basic nitrogen (TVBN) was calculated as mg TVBN/100 g fish.

### 2.8. Thiobarbituric Acid–Reactive Substances Content

In line with the method reviewed by Wang et al., the thiobarbituric acid–Reactive Substances (TBARS) value of fish meat was determined by the distillation method [23]. Specifically, 10.0 g of chopped fish abdominal meat was taken in a Kjeldahl distillation flask, 20 mL of deionized water was added and mixed well, then 2 mL of hydrochloric acid solution (mHCl: mH2O = 1:2) and 2 mL of liquid paraffin were added, and 50 mL of distillate was collected by hydrodistillation. 5 mL of distillate was mixed with 5 mL of TBARS acetic acid solution (0.2883 g of thiobarbituric acid dissolved in 100 mL of 90% glacial acetic acid) in a colorimetric tube, heated in a water bath at 100 °C for 35 min and then cooled to room temperature, and the absorbance A was measured at 535 nm. Distilled water was used as a blank sample instead of a distilled solution. The TBARS content was expressed as mg/kg fish meat.

### 2.9. Total Viable Count (TVC)

The total viable count was determined according to the Chinese national standard method GB 4789.2-2016 [24]. 5 g of ground fish was mixed with 45 mL of sterile saline (0.85 wt% NaCl), and then diluted with sterile saline in a 10-fold gradient to the appropriate concentration. The total number of colonies for each sample was determined by the pour plate technique using plate count agar (PCA) and incubated at 30 °C for 72 h. The colonies formed on each plate were counted, and the results were expressed as the logarithm of the number of colony-forming units per gram (log (CFU/g)).

### 2.10. Histological Analysis

As described in the study of Yu et al., samples were cut into 1.5 cm × 1.5 cm × 1.0 cm blocks, placed in 4% paraformaldehyde fixative for 24 h, then dehydrated, paraffin-embedded and sliced and stained with hematoxylin-eosin [11]. The microstructure of the fish was observed and photographed using a microscope with a scanner (Pannoramic MIDI, 3DHISTECH, Budapest, Hungary). The images shown were obtained at 200× magnification.

### 2.11. Biogenic Amine Content

Biogenic amines in fish were determined by high-performance liquid chromatography (HPLC) according to the Chinese national standard GB 5009.208-2016 [25]. The method involved dansulfonyl chloride derivatization. A Waters e2695 high-performance liquid chromatograph (Waters Milford, Milford, MA, USA), a Waters 2996 dual UV detector, an Empower system software (Version 3.9.0, Waters Corporation, Milford, MA, USA) and a Venusil C18 plus column (4.6 × 250 mm, 5 μm, Agela technologies, Tianjin, China) were used.

Specifically, 10.0 g of chopped fish was mixed with 20 mL of 5% trichloroacetic acid solution and shaken for 30 min, then centrifuged at 5000 r/min for 10 min, the supernatant was collected, and the residue was extracted again with 20 mL of 5% trichloroacetic acid solution. The supernatant was combined, and the final volume was adjusted to 50 mL with 5% trichloroacetic acid solution. 1 mL of supernatant was mixed with 1 mL of saturated sodium bicarbonate solution, 100 μL of sodium hydroxide solution (1 mol/L) and 1 mL of dansulfonyl chloride solution (10 mg/mL) and placed in a water bath at 60 °C for 15 min. 100 μL of sodium glutamate solution (50 mg/mL) was added for 15 min at 60 °C. After cooling to room temperature, 1 mL of deionized water was added and mixed well. The acetone was removed by nitrogen blowing at 40 °C in a water bath. Then 0.5 g of sodium chloride was added and vortexed to dissolve, 5 mL of ether was added, vortexed and shaken for 2 min, left to stratify, the upper organic phase (ether layer) was aspirated and extracted again, and the ether extract was combined and blown dry under nitrogen in a 40 °C water bath. 1 mL of acetonitrile was added to dissolve the residue, and filtered through a 0.22 μm membrane needle head filter and injected into the HPLC.

The gradient elution process was performed with ultrapure water as solvent A and acetonitrile as solvent B. The gradient elution procedure was performed as shown in Table 1 with a run time of 45 min. The flow rate was 1.0 mL/min, and 20 μL of sample was injected into the column. The column temperature was set at 30 °C, and the detection wavelength was 254 nm.

### 2.12. Statistical Analysis

The data in this manuscript were expressed as the mean ± standard deviation. Statistical analyses were performed by SPSS software version 26.0, and the results were analyzed using a one-way analysis of variance (ANOVA) and Duncan’s multiple range test to establish the significance of differences (*p* ≤ 0.05) among the mean values.

## 3. Results and Discussion

### 3.1. Water Content and Water Activity

**Figure 1:**
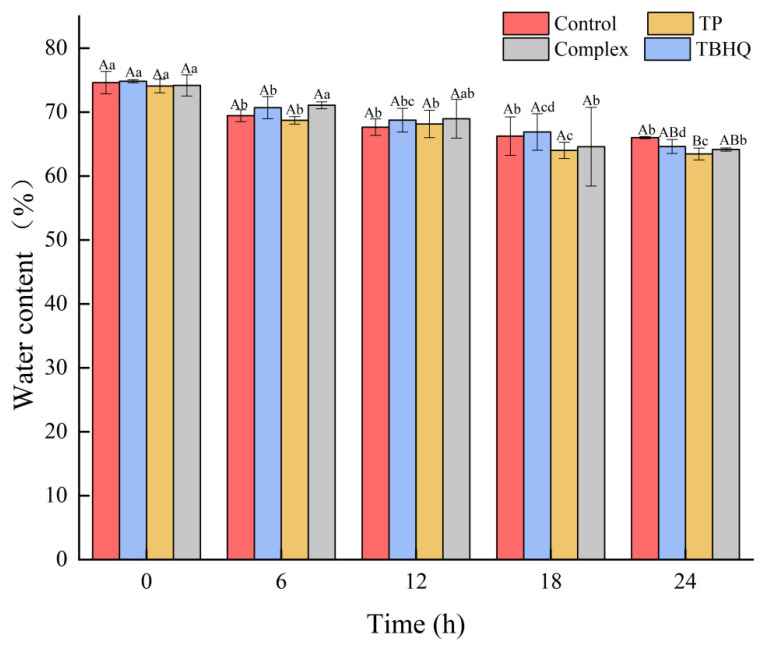
Changes in water content of topmouth culter during air drying. Different lowercase letters represent significant differences between the data in groups (*p* < 0.05). Different uppercase letters represent significant differences between the data of the same group at different time points (*p* < 0.05).

Fresh topmouth culter had an initial moisture content of 79.48%. After 12 h of salting, dehydration occurred, reducing the moisture level by approximately 5%. Figure 1 illustrates the evolution of moisture content in fish salted with salt and antioxidants during air drying at 25 °C. As drying progressed, the moisture content progressively declined, with a particularly pronounced drop at 6 h, followed by a slower decreasing trend. At 24 h of air drying, the moisture content of the samples ranged from 63.45% to 66.01%, representing an overall reduction of approximately 10%. No significant differences (*p* < 0.05) in water content were observed among the four groups of samples. This is consistent with previous reports that slightly salted silver carp filets were dried at 15 °C with cold air, where the rate of migration of water molecules from the interior of the fish to the surface was greater than the rate of evaporation of water from the surface during the early stages of drying, and thus the rate of moisture reduction was faster in the early stages of drying. Similar to the findings of Yu et al. on silver carp, our study showed a progressive decrease in moisture content during drying. However, unlike Yu et al., who compared cold and hot air drying, we employed a combined cold smoking and air-drying process with antioxidant pretreatment [11]. However, as the drying progressed, the drying rate decreased due to the surface shrinkage of the samples as well as the decrease in water content. This phenomenon occurs because surface shrinkage forms a compact outer layer that hinders internal moisture migration, while the reduced moisture gradient between the interior and the surface further lowers the drying rate [26].

**Figure 2:**
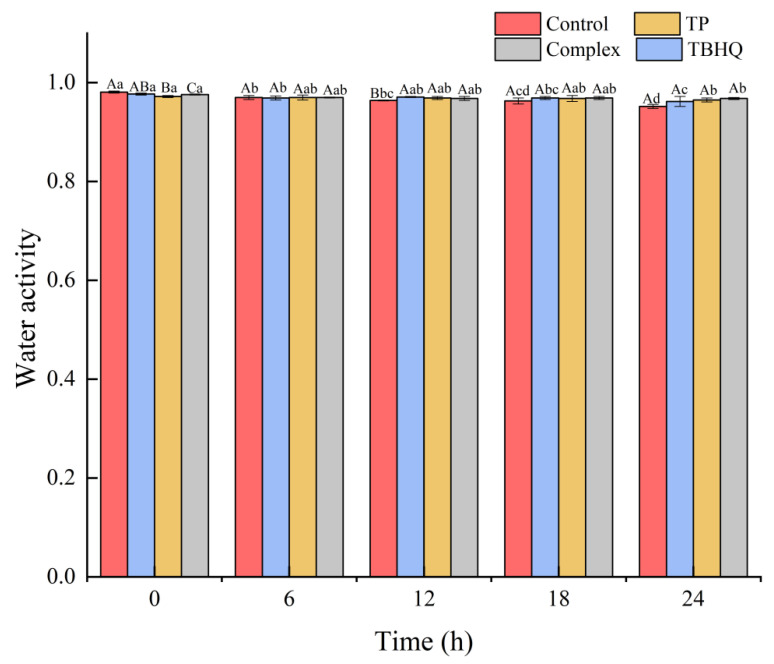
Changes in water activity of topmouth culter during air drying. Different lowercase letters represent significant differences between the data in groups (*p* < 0.05). Different uppercase letters represent significant differences between the data of the same group at different time points (*p* < 0.05).

The changes in water activity of fish meat with time during the air-drying process are shown in Figure 2. Water activity showed a decreasing trend with the increase in air-drying time, which was positively correlated with water content (Figure 1). The reduction in water activity is primarily attributed to the removal of free water, which increases the concentration of solutes (e.g., salts and other low-molecular-weight compounds) in the remaining water phase, thereby lowering the vapor pressure and water activity [26]. Additionally, as drying progresses, protein denaturation and structural shrinkage may reduce the availability of water-binding sites, further contributing to the decline in water activity [11]. The water activity of the control group decreased from 0.981 to 0.952. For the antioxidant-treated groups (TBHQ, TP, and complex), water activity followed a similar decreasing trend but showed no significant change between 18 h and 24 h, suggesting a slightly faster stabilization of water activity in the presence of antioxidants. However, overall differences in aw between the control and antioxidant-treated groups were not statistically significant (*p* > 0.05). This indicates that the addition of 0.2 wt% TBHQ and/or TP does not markedly influence the moisture retention or water-binding capacity of the fish muscle during air drying. This finding can be explained by the fact that TBHQ is lipophilic and primarily partitions into lipid phases rather than interacting with water or protein hydrophilic groups [15], while TP at the tested concentration (0.2 wt%) is likely insufficient to induce significant protein crosslinking that would alter water binding [13,27].

### 3.2. Water Distribution

The measurement of T_2_ relaxation can reflect the changes in the water distribution of the sample during drying. T_2b_ with low relaxation times (<10 ms) is classified as water tightly bound to macromolecules, T_21_ with relaxation times of 10–100 ms is considered as immobile water, and T_22_ (100–1000 ms) represents free water [19]. Table 2 summarizes the proportion of water in the three states and the change in relaxation time during the air drying of fish. As the drying time increased, both T_2b_ and T_21_ shifted to the left, and the proportion of bound water (P_2b_) increased while that of immobile water (P_21_) decreased. This shift indicates that water molecules became more tightly associated with macromolecules due to the removal of free water and the concentration of solutes, which enhances water–protein interactions [19]. Concurrently, the progressive dehydration causes myofibrillar proteins to denature and aggregate, reducing the space available for entrapped immobile water and converting some immobile water into bound water [11,28]. The percentage of immobile water in the fish ranged from 93.87% to 94.58% after 24 h of air drying, remaining the predominant water component throughout the process. From a quality perspective, the increase in bound water (P_2b_) and the reduction in T_21_ relaxation time are associated with a denser muscle structure, which contributes to improved hardness and chewiness and may enhance the storage stability of the product by reducing the availability of free water for microbial growth [4]. The observed T_22_ peak (free water) remained very low (<1.3%) in all samples, confirming that the drying process effectively removed most free water, which is a key factor in preserving fish quality [11].

**Table 2:** Changes in relaxation time and fraction of each relaxation component during the air drying.

Group	Time (h)	T_2b_ (ms)	T_21_ (ms)	T_22_ (ms)	P_2b_ (%)	P_21_ (%)	P_22_ (%)
Control	0	2.98 ± 0.16 ^a^	55.51 ± 0.00 ^a^	938.62 ± 18.65 ^b^	3.77 ± 0.42 ^c^	96.07 ± 0.41 ^a^	0.16 ± 0.02 ^b^
	6	1.24 ± 0.14 ^c^	39.79 ± 2.82 ^b^	1174.59 ± 142.82 ^a^	3.38 ± 0.64 ^c^	96.53 ± 0.65 ^a^	0.09 ± 0.01 ^b^
	12	2.08 ± 0.32 ^b^	38.88 ± 2.84 ^b^	690.94 ± 155.99 ^c^	5.05 ± 0.82 ^b^	94.78 ± 0.81 ^b^	0.17 ± 0.15 ^b^
	18	1.90 ± 0.46 ^b^	31.66 ± 3.42 ^c^	469.61 ± 9.33 ^d^	6.25 ± 0.48 ^a^	93.56 ± 0.47 ^c^	0.19 ± 0.06 ^b^
	24	0.58 ± 0.17 ^d^	22.59 ± 4.23 ^d^	268.04 ± 89.92 ^e^	4.17 ± 0.42 ^bc^	94.58 ± 0.72 ^bc^	1.25 ± 0.80 ^a^
TBHQ	0	1.53 ± 0.31 ^cd^	55.58 ± 3.00 ^a^	975.11 ± 211.71 ^a^	2.99 ± 0.39 ^c^	96.89 ± 0.41 ^a^	0.12 ± 0.07 ^ab^
	6	3.66 ± 0.28 ^a^	41.36 ± 1.01 ^b^	0.00 ± 0.00 ^c^	4.60 ± 0.59 ^b^	95.40 ± 0.59 ^b^	0.00 ± 0.00 ^b^
	12	2.66 ± 0.83 ^ab^	36.14 ± 4.41 ^c^	827.09 ± 40.48 ^a^	4.42 ± 0.02 ^b^	95.40 ± 0.02 ^b^	0.28 ± 0.00 ^ab^
	18	2.26 ± 0.79 ^bc^	33.18 ± 4.11 ^c^	433.77 ± 132.12 ^b^	5.59 ± 0.50 ^a^	93.61 ± 0.59 ^c^	0.80 ± 0.80 ^a^
	24	1.14 ± 0.38 ^d^	23.77 ± 0.58 ^d^	287.55 ± 7.04 ^b^	5.66 ± 0.18 ^a^	93.87 ± 0.09 ^c^	0.46 ± 0.09 ^ab^
TP	0	2.16 ± 0.42 ^a^	57.04 ± 6.15 ^a^	576.96 ± 149.50 ^b^	3.35 ± 0.93 ^a^	96.39 ± 0.79 ^a^	0.26 ± 0.13 ^c^
	6	1.43 ± 0.13 ^b^	44.58 ± 0.89 ^b^	1241.15 ± 107.13 ^a^	3.38 ± 0.05 ^a^	96.51 ± 0.03 ^a^	0.11 ± 0.02 ^bc^
	12	2.53 ± 0.20 ^a^	33.85 ± 2.48 ^c^	476.35 ± 44.55 b^c^	4.30 ± 0.81 ^a^	95.47 ± 0.77 ^ab^	0.23 ± 0.07 ^bc^
	18	1.37 ± 0.24 ^b^	30.48 ± 1.23 ^c^	400.19 ± 112.26 ^cd^	4.30 ± 0.61 ^a^	95.19 ± 0.80 ^ab^	0.51 ± 0.26 ^b^
	24	0.41 ± 0.01 ^c^	16.74 ± 1.19 ^d^	232.46 ± 45.70 ^d^	4.81 ± 0.83 ^a^	94.06 ± 1.05 ^b^	1.13 ± 0.24 ^a^
Complex	0	2.68 ± 0.36 ^ab^	54.88 ± 1.09 ^a^	964.45 ± 110.20 ^a^	3.62 ± 0.34 ^a^	96.10 ± 0.17 ^a^	0.28 ± 0.18 ^bc^
	6	3.26 ± 1.04 ^a^	42.82 ± 1.05 ^b^	1091.07 ± 53.41 ^a^	4.40 ± 0.22 ^a^	95.48 ± 0.07 ^a^	0.12 ± 0.05 ^c^
	12	2.29 ± 1.68 ^ab^	35.02 ± 1.84 ^c^	717.04 ± 170.17 ^b^	5.22 ± 2.15 ^a^	94.58 ± 2.11 ^a^	0.21 ± 0.07 ^bc^
	18	1.54 ± 0.42 ^ab^	26.61 ± 2.73 ^d^	269.86 ± 5.36 ^c^	4.50 ± 0.31 ^a^	94.66 ± 0.48 ^a^	0.84 ± 0.22 ^a^
	24	1.18 ± 0.28 ^b^	24.19 ± 9.43 ^d^	299.88 ± 21.93 ^c^	5.12 ± 0.94 ^a^	94.43 ± 0.97 ^a^	0.45 ± 0.07 ^b^

Data are presented as mean ± standard deviation (n = 3). T_2b_: bound water relaxation time; T_21_: immobile water relaxation time; T_22_: free water relaxation time; P_2b_: proportion of bound water (%); P_21_: proportion of immobile water (%); P_22_: proportion of free water (%). Different superscript letters within the same column and the same treatment group indicate significant differences (*p* < 0.05) as determined by one-way ANOVA followed by Duncan’s multiple range test.

### 3.3. Color Measurement

The color of fish products is an important sensory attribute that can be used to assess the effect of the drying method on the appearance of the fish. Table 3 shows the changes in brightness (L*), redness (a*) and yellowness (b*) values of the fish during drying time. As the air-drying time increased, the brightness of the fish decreased continuously, the redness value increased slowly, and the yellowness value increased significantly. The brightness of the control samples decreased from 41.69 to 36.47. The addition of antioxidants slightly mitigated the reduction in L values, with the complex antioxidant group showing a decrease from 43.27 to 38.97. The color change in fish meat was correlated with the change in water content and distribution in the muscle [20]. In the later stages of air drying, the fish showed a yellowish-brown color, and the browning of the fish was attributed to the Maillard reaction and the oxidation of proteins and fats [4]. Regarding the effect of antioxidants, both TBHQ and TP effectively inhibited the increase in redness (a*) compared to the control, which is attributed to their ability to retard lipid oxidation and stabilize heme pigments [13,29]. TP has been reported to inhibit myoglobin oxidation through non-covalent binding, thereby preserving the red color of fish muscle during storage [30]. Similarly, TBHQ suppresses the formation of colored secondary oxidation products by scavenging free radicals and chelating metal ions involved in heme-mediated lipid oxidation [31].

**Table 3:** Changes in brightness (L*), redness (a*) and yellowness (b*) values of the fish with drying time during the air-drying process.

Color	Time (h)	Control	TBHQ	TP	Complex
L*	0	41.69 ± 1.70 ^a^	40.75 ± 0.61 ^a^	35.53 ± 0.56 ^a^	43.27 ± 1.03 ^a^
	6	40.66 ± 1.15 ^b^	38.67 ± 0.52 ^b^	34.82 ± 0.64 ^b^	38.59 ± 0.97 ^b^
	12	39.53 ± 0.99 ^c^	35.84 ± 0.83 ^c^	34.56 ± 0.35 ^b^	36.68 ± 0.39 ^c^
	18	37.52 ± 0.20 ^d^	35.09 ± 0.54 ^cd^	33.70 ± 0.35 ^c^	34.60 ± 0.60 ^d^
	24	36.47 ± 0.81 ^e^	34.42 ± 0.73 ^d^	32.45 ± 2.13 ^cd^	32.40 ± 0.78 ^e^
a*	0	−2.37 ± 1.08 ^e^	−3.75 ± 0.14 ^e^	−3.62 ± 0.71 ^e^	−3.69 ± 0.34 ^d^
	6	−1.10 ± 0.07 ^d^	−1.56 ± 0.36 ^d^	−1.78 ± 0.06 ^d^	−1.26 ± 0.52 ^c^
	12	0.44 ± 0.58 ^c^	−0.61 ± 0.54 ^c^	−0.59 ± 0.16 ^c^	0.11 ± 0.20 ^b^
	18	1.25 ± 0.10 ^b^	0.25 ± 0.01 ^b^	0.38 ± 0.34 ^b^	0.80 ± 0.14 ^a^
	24	2.39 ± 0.73 ^a^	0.40 ± 0.14 ^a^	0.71 ± 0.12 ^a^	0.82 ± 0.09 ^a^
b*	0	−0.26 ± 0.59 ^e^	1.50 ± 0.27 ^e^	0.40 ± 0.17 ^e^	2.45 ± 0.37 ^d^
	6	4.37 ± 1.29 ^d^	4.99 ± 0.69 ^d^	4.47 ± 0.85 ^d^	5.75 ± 1.21 ^c^
	12	6.30 ± 1.07 ^c^	6.55 ± 0.62 ^c^	6.42 ± 0.09 ^c^	6.94 ± 0.80 ^c^
	18	8.12 ± 0.11 ^b^	7.27 ± 0.72 ^b^	7.76 ± 0.54 ^b^	8.25 ± 1.56 ^b^
	24	9.19 ± 1.32 ^a^	8.01 ± 0.16 ^a^	9.16 ± 1.31 ^a^	10.38 ± 0.70 ^a^

Data are presented as mean ± standard deviation (n = 5). L*: brightness (0 = black, 100 = white); a*: redness (positive = red, negative = green); b*: yellowness (positive = yellow, negative = blue). Different superscript letters within the same column and the same color parameter indicate significant differences (*p* < 0.05) as determined by one-way ANOVA followed by Duncan’s multiple range test.

### 3.4. Texture Analysis

The textural properties of fish meat can reflect the degree of internal muscle tissue denseness. Table 4 shows the changes in hardness and chewiness of fish meat with air-drying time. The hardness of the fish meat increased significantly with increasing air-drying time, and the chewiness also increased. At 24 h of air-drying, the hardness of the control group increased by 1.3 times, and the hardness of the fish salted with TBHQ, TP, and complex antioxidants increased by 1.5, 2.0, and 1.37 times, respectively. The chewiness of fish with 24 h of air-drying was three times higher than the chewiness of the non-air-dried fish. This was due to the decreasing moisture content of the fish and the denaturation and contraction of myogenic fibrous proteins [21]. This is in agreement with previous reports that after 30 h of drying using cold air (15 °C), the hardness values of fish meat increased by 2.41 times, mainly attributed to surface hardening caused by reduced moisture [11]. There was no significant effect of antioxidant addition on fish texture during the air-drying process. This lack of effect is expected, as antioxidants primarily target lipid oxidation rather than protein structural changes that govern texture. The observed texture changes are therefore mainly attributed to moisture loss and protein denaturation, which occur independently of antioxidant treatment [21].

**Table 4:** Changes in the hardness (g) and chewiness (g) of fish during air drying.

Texture	Time (h)	Control	TBHQ	TP	Complex
Hardness	0	1077.7 ± 32.5 ^e^	932.0 ± 55.2 ^d^	719.7 ± 75.7 ^e^	937.0 ± 73.3 ^e^
	6	1646.5 ± 92.7 ^d^	1584.0 ± 44.0 ^c^	1510.2 ± 58.8 ^d^	1520.1 ± 128.3 ^d^
	12	1850.0 ± 129 ^c^	1628.3 ± 95.5 ^c^	1830.9 ± 105.0 ^c^	1876.6 ± 17.2 ^c^
	18	1937.4 ± 135.9 ^b^	1910.0 ± 64.0 ^b^	2011.4 ± 57.0 ^b^	2046.2 ± 75.9 ^b^
	24	2475.8 ± 65.6 ^a^	2304.4 ± 123.0 ^a^	2197.8 ± 33.0 ^a^	2224.6 ± 65.7 ^a^
Chewiness	0	279.8 ± 15.5 ^e^	162.9 ± 11.8 ^d^	191.0 ± 17.9 ^e^	152.4 ± 9.1 ^e^
	6	300.7 ± 39.7 ^d^	372.3 ± 13.2 ^c^	232.1 ± 8.0 ^d^	227.5 ± 23.5 ^d^
	12	319.0 ± 125.6 ^c^	379.8 ± 3.0 ^c^	347.2 ± 12.4 ^c^	294.5 ± 16.5 ^c^
	18	494.1 ± 64.5 ^a^	420.0 ± 1.7 ^b^	407.0 ± 15.7 ^b^	404.2 ± 36.2 ^b^
	24	462.4 ± 35.4 ^b^	487.8 ± 57.1 ^a^	508.7 ± 48.3 ^a^	441.4 ± 31.4 ^a^

Data are presented as mean ± standard deviation (n = 5). Hardness and chewiness are textural parameters measured using a texture analyzer in TPA mode. Different superscript letters (a, b, c, d, e) within the same column and the same texture parameter indicate significant differences (*p* < 0.05) as determined by one-way ANOVA followed by Duncan’s multiple range test.

### 3.5. Total Volatile Basic Nitrogen

**Figure 3:**
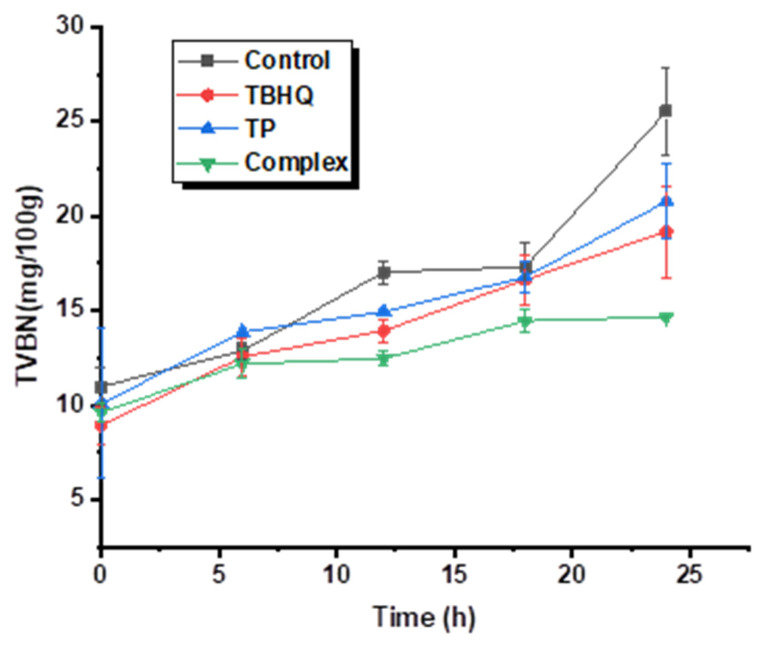
Effects of different antioxidants on the TVBN value of air-drying fish.

The total volatile basic nitrogen (TVBN) is an important physicochemical index for the evaluation of the freshness of aquatic products. The TVBN content in fresh topmouth culter was 8.68 mg/100 g. After 12 h of salting, the content increased slightly. Figure 3 shows the changes in TVBN content in the samples with drying time. The TVBN content in all four groups of samples showed an increasing trend as the air-drying time increased. This increase was closely related to the activity of spoilage bacteria and endogenous enzymes [22]. The addition of antioxidants had an inhibitory effect on the increase in TVBN content in fish during the air-drying process, and the inhibition was particularly significant for fish salted with a complex of antioxidants. The superior performance of the complex antioxidant group suggests a synergistic effect between TBHQ and TP in suppressing microbial deamination of non-protein nitrogen compounds [22]. This finding is consistent with previous reports that combining synthetic and natural antioxidants can enhance spoilage inhibition in dried fish products [4]. The TVBN content of control was 25.53 mg/100 g at 24 h of air-drying, which was 2.5 times higher than the TVBN content in fish without air-drying treatment, while the TVBN content of the complex antioxidant was only 14.66 mg/100 g at 24 h of air-drying, which was lower than the TVBN content in TBHQ or TP salted fish. This was attributed to the ability of the presence of TBHQ and TP to reduce the ability of bacteria to oxidatively de-aminate non-protein nitrogen compounds [23]. In a previous study, Song, Liu, Shen, You and Luo used an alginate edible coating containing tea polyphenols to inhibit the increase in TVBN content of refrigerated bream (Megalobrama amblycephala) during storage, thereby extending its shelf life [32].

### 3.6. Thiobarbituric Acid-Reactive Substances

**Figure 4:**
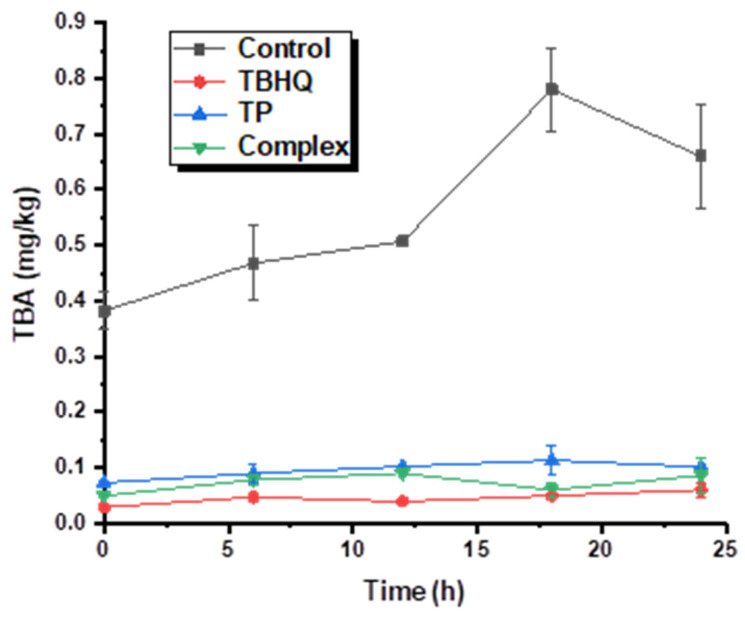
Effects of different antioxidants on the TBARS value of air-drying fish.

The TBARS value is widely used as an indicator to evaluate the degree of lipid oxidation in meat products and reflects the secondary products of lipid oxidation by hydroperoxides. In Figure 4, the TBARS values of the control samples showed a trend of significant increase followed by a slight decrease with increasing drying time. However, the TBARS values of fish salted with antioxidants did not increase significantly (*p* < 0.05) during air-drying. The TBARS content of the control group increased from 0.38 mg/kg to 2.64 mg/kg from 0 h to 24 h. The TBARS content of the other three groups was lower than 0.10 mg/kg at 24 h. This indicates that the addition of antioxidants was effective in inhibiting fat oxidation in the fish during air-drying. In addition, the TBARS content of the fish marinated with TBHQ was consistently lower than that of the other groups during air-drying. The superior efficacy of TBHQ is attributed to its strong free radical scavenging activity and its lipophilic nature, which allows it to partition into the lipid phase where oxidation primarily occurs [15]. This is in agreement with Xu et al., who reported that TBHQ effectively suppressed lipid oxidation in fermented fish paste. It was reported that TBARS content in fish paste remained low when 0.03% TBHQ was added to the fermented fish paste, which proved that TBHQ effectively inhibited lipid oxidation in the fish paste [29].

### 3.7. Total Viable Count (TVC)

**Figure 5:**
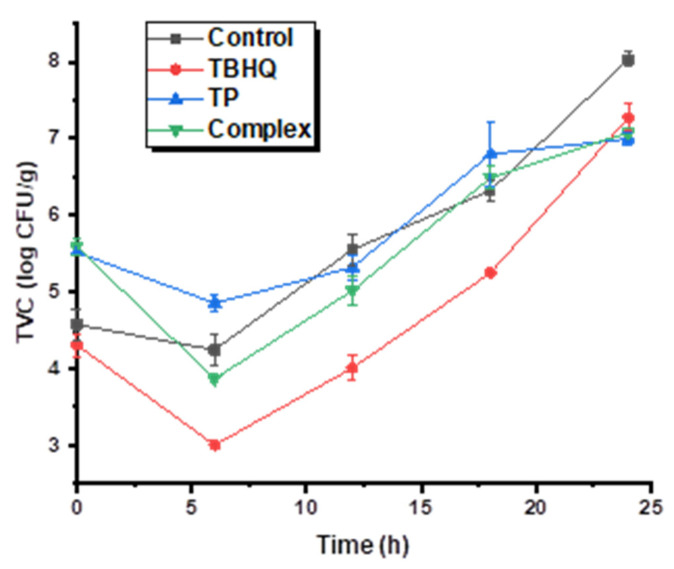
Effects of different antioxidants on the TVC of air-drying fish.

The total viable count (TVC) was determined to assess the changes in microorganisms during air drying. As shown in Figure 5, the TVC of the four groups of samples without air-drying were 4.57, 4.31, 5.52 and 5.59 log CFU/g. During the air-drying process, the TVC in the fish showed a decrease followed by an increase. At 6 h of air-drying, the TVC was the lowest in the four groups of samples, which was due to the inhibition of microbial growth caused by the significant decrease in the moisture content of the fish at the beginning of air-drying. With the increase in air-drying time, the TVC increased, and at 24 h, the TVC of the four groups increased by 3.47, 2.96, 1.48, and 1.49 log CFU/g, respectively, indicating that the addition of antioxidants inhibited the increase in microbes during the air-drying process. Previous studies have shown that 0.2% TP dip treatment inhibited the growth of spoilage bacteria during frozen storage of silver carp [9]. The antimicrobial activity of TP is primarily attributed to its ability to disrupt bacterial cell membranes and chelate metal ions essential for microbial growth [13]. In addition, TP has a synergistic antibacterial effect with kojic acid for frozen sea bass (*Lateolabrax japonicus*) filets, thus increasing the shelf life of the fish [23].

### 3.8. Histological Analysis

**Figure 6:**
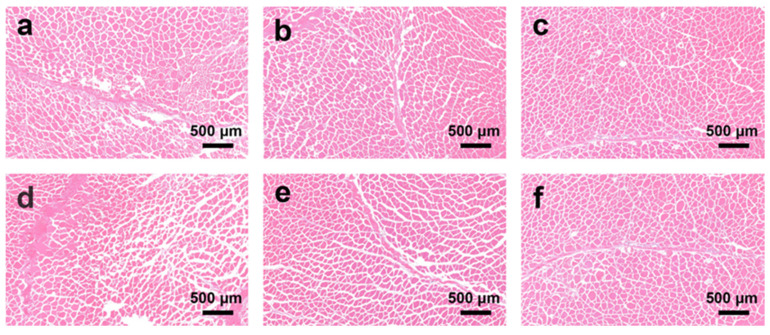
Histological observations of fish treated without antioxidant (**a**–**c**) and treated with complex antioxidants (**d**–**f**) during air-drying at 0 h (**a**,**d**), 12 h (**b**,**e**) and 24 h (**c**,**f**).

Figure 6 demonstrates the changes in the microstructure of the fish during the air-drying process. The muscles of non-air-dried fish have more gaps between muscle tissues and unevenness. As the air-drying time increased, the gaps in the cross-section of the fish became smaller, and the fish structure showed a contracted and tight structural feature, which was mainly attributed to the water loss of muscle fiber proteins, and the muscle tissue became dense [33]. At 24 h of air drying, the muscle tissue of the fish was firm and homogeneous. The addition of antioxidants did not have a significant effect on the microstructure of fish. This observation is consistent with the texture analysis results (Section 3.4), confirming that antioxidants do not interfere with the physical densification caused by moisture loss. The compact microstructure observed after 24 h of drying is favorable for product stability, as it reduces oxygen penetration and limits microbial access to nutrients [21]. It has been reported that different drying methods have a great influence on the density of muscle fibers [34,35]. High temperature drying was prone to be associated with the destruction of the histological structure of the proteins of fish flesh, leading to the collapse of the muscle tissue structure [36]. It is consistent with our findings that air-dried fish meat has a more compact microstructure, as reported by Pankyamma, Mokam, Debbarma and Rao [21].

### 3.9. Biogenic Amine Content

**Figure 7:**
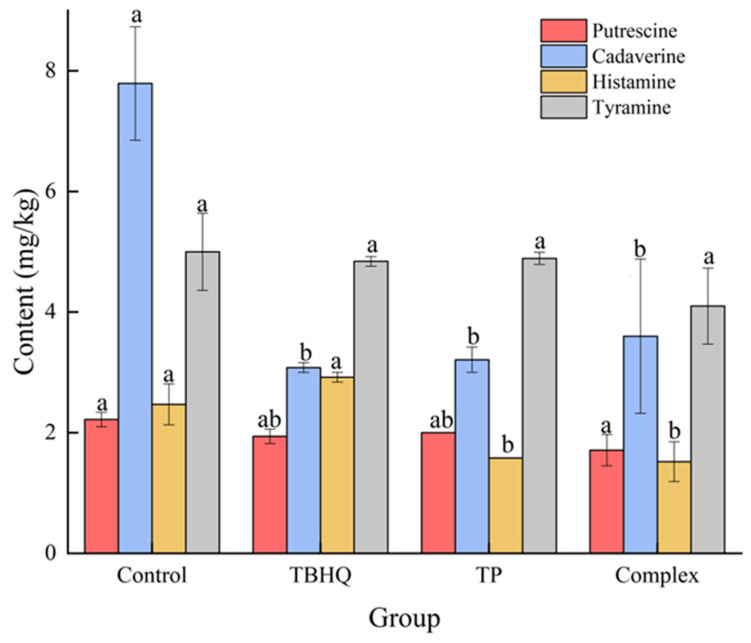
The biogenic amine content in fish after 24 h of air drying. Different lowercase letters represent significant differences between the data in groups (*p* < 0.05).

Biogenic amines are widely concerned because of their adverse health effects [37]. Cadaverine, histamine and putrescine are important compounds related to fish safety, and they can be used as indicators for the evaluation of fish freshness [38]. The biogenic amine content in the air-dried fish for 24 h is shown in Figure 7. The levels of cadaverine, histamine, tyramine and putrescine all remained low at 24 h of air-drying, indicating that the fish was of better quality at this time. The levels of cadaverine and putrescine in the other three groups of samples salted with antioxidants were lower than the levels of biogenic amines in the control group. The levels of putrescine in the antioxidant salted air-dried fish ranged from 1.71 to 2.00 mg/kg, the levels of cadaverine were between 3.08 and 3.21 mg/kg, the levels of tyramine were between 4.10 and 4.89 mg/kg, and the levels of histamine were between 1.52 and 2.92 mg/kg, which were well below their safety limits. This meant that the antioxidants inhibited the production of biogenic amines in the fish during the air-drying process and ensured the freshness of the fish. The reduction in biogenic amine accumulation is likely due to the combined effects of antioxidant-mediated inhibition of spoilage bacteria (as evidenced by lower TVC) and reduced availability of free amino acids resulting from decreased proteolysis [38]. The levels of histamine (1.52–2.92 mg/kg) and tyramine (4.10–4.89 mg/kg) in all antioxidant-treated groups were well below the safety limits, indicating that the dried fish products are safe for consumption [37].

## 4. Conclusions

In conclusion, the addition of 0.2 wt% antioxidants (TP alone, TBHQ alone, or their combination) effectively preserved the quality of topmouth culter during air drying at 25 °C for 24 h. The drying process led to a reduction in moisture content (from 79.48% to approximately 64–66%) and water activity, accompanied by an increase in bound water and a denser muscle structure, which resulted in significantly increased hardness and chewiness. Color changes included decreased brightness and increased redness and yellowness. Importantly, all three antioxidant treatments significantly inhibited lipid oxidation (reflected by lower TBA values), reduced protein spoilage (lower TVBN), suppressed microbial growth (lower TVC), and decreased biogenic amine accumulation compared to the control. These findings demonstrate that the incorporation of TP, TBHQ, or their combination at 0.2 wt% during salting is an effective strategy to improve the quality and safety of salted air-dried topmouth culter, providing a practical basis for industrial application.

## Figures and Tables

**Table 1:** Gradient elution procedure.

Time (min)	0	10	15	20	27	30	35	40	45
Mobile phase A (%)	65	65	75	80	85	90	95	65	65
Mobile phase B (%)	35	35	25	20	15	10	5	35	35

## Data Availability

The data that has been used is confidential.
